# High-level psychotropic polypharmacy: a retrospective comparison of children in foster care to their peers on Medicaid

**DOI:** 10.1186/s12888-021-03309-9

**Published:** 2021-06-10

**Authors:** Deborah Winders Davis, W. David Lohr, Yana Feygin, Liza Creel, Kahir Jawad, V. Faye Jones, P. Gail Williams, Jennifer Le, Marie Trace, Natalie Pasquenza

**Affiliations:** 1 Department of Pediatrics, Child and Adolescent Health Research Design and Support Unit, University of Louisville, 571 S. Floyd Street, KY 40202 Louisville, USA; 2 Department of Health Management & System Sciences, University of Louisville School of Public Health and Information Science, 485 E. Gray Street, Louisville, KY 40202 USA

**Keywords:** Children, Psychotropic medications, Polypharmacy, Medicaid, Foster care

## Abstract

**Background:**

The use of antipsychotic medication and psychotropic polypharmacy has increased in the United States over the last two decades especially for children from low-income families and those in foster care. Although attention has been paid to providing greater insight, prescribing patterns remain concerning since there is a lack of evidence related to safety and efficacy. High-level psychotropic polypharmacy has not been described. We aim to compare the use of HLPP for children receiving Medicaid services and those in foster care and identify factors associated with the duration of use of high-level psychotropic polypharmacy. Additionally, we will examine the frequency of laboratory metabolic screening and emergency department, inpatient, and outpatient visits.

**Methods:**

A cross-sectional, secondary analysis of statewide data describes trends in high-level psychotropic polypharmacy from 2012 to 2017 and the prevalence and predictors of high-level psychotropic polypharmacy duration and resource use in 2017 for all children on Medicaid and those in foster care. High-level psychotropic polypharmacy included concurrent use, at least four classes of medications including an antipsychotic, and at least 30 days duration.

**Results:**

High-level psychotropic polypharmacy increased from 2012 to 2014 for both groups but stabilized in 2015–2016. Children in foster care showed a slight increase compared to their peers in 2017. There was no association between duration and demographic characteristics or foster care status. Diagnoses predicted duration. Neither group received metabolic monitoring at an acceptable rate.

**Conclusions:**

Concerning patterns of high-level psychotropic polypharmacy and metabolic monitoring were identified. Cautious use of high-level psychotropic polypharmacy and greater oversight to ensure that these children are receiving comprehensive services like behavioral health, primary care, and primary prevention.

**Supplementary Information:**

The online version contains supplementary material available at 10.1186/s12888-021-03309-9.

Antipsychotic medication use for children has increased substantially over the past 20–25 years, especially for children who are publicly insured [1–4]. Also on the rise is the use of other psychotropic medications concurrently with antipsychotic medications [5–8]. While psychotropic polypharmacy is increasingly common, little data exists to support its safety and efficacy in children and youth [5, 7]. Krieger and colleagues [5] found that second-generation antipsychotic medication use increased from 2004 to 2008 along with the proportion of polypharmacy using other classes of psychotropic medication with the rates of concurrent use being 30 to 60% higher for children in foster care compared to other publicly insured children [5]. Based on these data, there has been a push to provide more oversight for children receiving psychotropic medications especially those in the foster care system [9–12].

Kentucky ranks among the highest U.S. states for rates of psychotropic medication use for children, including antipsychotic medications [12]. Children in foster care are at great risk for behavioral and mental health problems due to trauma, stress, and exposure to numerous adverse childhood events [13–19]. In complex cases, psychotropic polypharmacy may be needed and appropriate. However, there is little evidence supporting the safety and efficacy of psychotropic polypharmacy, especially high-level psychotropic polypharmacy (HLPP) in children and adolescents [7, 20–22]. Similarly, more data are needed to better understand the long-term effects of early psychotropic medication use on the development of brain structure and function [23–27]. A recent systematic review found that the literature has been inconsistent in defining pediatric polypharmacy, in general [28]. Consequently, the authors recommended that all studies of polypharmacy specify the number of medications or classes of medications, whether the medications are concurrent, and the duration [28]. We defined psychotropic polypharmacy as the concurrent use of at least two classes of psychotropic medications, while high-level psychotropic polypharmacy was the concurrent use of at least four classes of psychotropic medications for at least 30 days during the calendar year. We found no previous definitions of HLPP.

Psychotropic medications are expensive and have many side effects, some of which bear a long-term burden on health and finances [20, 29–31]. Some of the known side effects include increases in body mass indexes, obesity, glucose dysregulation, hyperlipidemia, type 2 diabetes mellitus, and fatigue/somnolence [20, 30–33]. Care must be taken to ensure that those children requiring antipsychotic medication and polypharmacy are appropriately followed to monitor for metabolic dysfunction and to assess ongoing symptoms and potential side effects [33–36]. The concern for metabolic dysfunction is even greater for children in the foster care system and those suffering from other traumas and toxic stress as these factors may compound the damaging effects of the medications on metabolic systems [37, 38]. Caution is warranted for all psychotropic medication use, especially HLPP and antipsychotics.

Kentucky ranks eighth in the U. S for the percent of children (43%) receiving Medicaid services compared to the national average of 37.5%. Just under one-half percent of the state’s children are in foster care, which is slightly lower than the national average.

The current study aims to describe HLPP use and factors associated with the duration of HLPP use in children receiving Kentucky Medicaid and those in foster care in 2017. These data are more recent than those previously reported in the literature and HLPP duration has not been reported. The following section includes operational definitions for the current study.

## Methods

### Data source and sample

A dataset of diagnostic, pharmaceutical, and demographic information was compiled using Kentucky Medicaid enrollment and billing claims data from 1/1/2012 through 12/31/2017. The data were provided by the Kentucky Cabinet for Health and Family Services, Department of Medicaid Services. The medical and pharmaceutical billing claims include information about service date, prescription, type of claim, postal codes for provider and enrollee, provider, and associated International Classification of Diseases, 9th ed. [ICD-9] code and ICD-10 code, while the enrollment information was used to add patient demographic information such as age, sex, foster care status, and self-identified race for all enrollees. To describe the epidemiology of and factors associated with the duration of high-level psychotropic polypharmacy (HLPP) in children, an analytic cohort of children between 6 and 17 years of age during 2017 was created. The children in the cohort were without a seizure or convulsion diagnosis (See Supplemental Table S1 for ICD codes), continuously enrolled throughout 2017, had at least one claim for an antipsychotic medication (APM), and were treated with HLPP for at least 30 days in the calendar year (*n* = 2240). Previous studies of polypharmacy used receipt of at least one antipsychotic medication in the inclusion criteria [5, 39]. Children were stratified between those within the foster care system (*n* = 417), and those enrolled in Medicaid, but not in foster care (*n* = 1823). Trends in HLPP are described for the years 2012–2017.

**Fig. 1:**
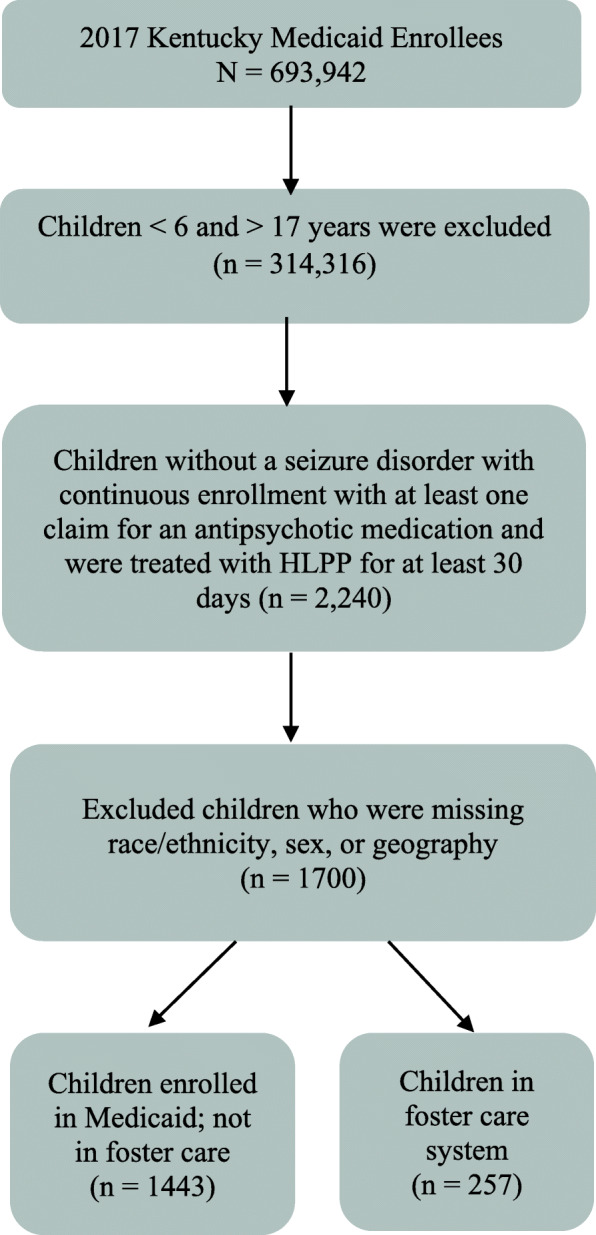
Description of Sample Inclusions/Exclusions for High-level Psychotropic Polypharmacy (HLPP) in 2017 Kentucky Medicaid Claim Data

A secondary data set was created for the regression analysis, investigating the risk factors associated with a longer duration of HLPP among children in our sample population. Here, we excluded children with incomplete records of race/ethnicity, sex, or geography, reducing our sample to 257 children in foster care and 1443 children receiving Medicaid services who are not in foster care (Fig. 1).

### Definition of utilization, psychotropic medications, and diagnostic categories

The presence of relevant ICD-10 codes for psychiatric disorders in any of the four available diagnostic code columns in any of the medical billing claims was used to establish diagnostic information for the analytic sample. Several common psychiatric comorbidities were included as predictors in our HLPP duration model, categorized as follows: (1) Disruptive behavior disorders, (2) Impulse control disorders, (3) Anxiety and trauma disorders, (4) Autism disorders, (5) Mood disorders, (6) Other neurodevelopmental disorders, (7) Schizophrenia and other psychoses, and (8) Sleep-wake disorders. For a full description of diagnoses that were used and ICD codes, please see Supplemental Table S1.

Utilization of services such as outpatient visits, inpatient admissions and emergency department visits were defined using Current Procedural Terminology (CPT) codes within the medical billing claims. CPT codes indicating each type of visit were summed over the year for each patient and presented as mean number of visits in Table 2. Metabolic screening was defined as the presence of both lipid and glucose lab testing, as indicated by CPT codes. It has been recommended that children on antipsychotic medication receive at least annual monitoring.

Classes of dispensed psychiatric medications were identified using National Drug Codes (NDC) within the patient pharmacy claims. These were categorized as: (1) antidepressants, (2) anxiolytics, (3) antipsychotics, (4) stimulants and atomoxetine, (5) mood stabilizers, (6) alpha-agonists, and (7) lithium. For a full description of medications that were used, please see Supplemental Table S2.

### Definition of high-level psychotropic polypharmacy and psychotropic polypharmacy

High-level psychotropic polypharmacy was defined as the concurrent use of at least four classes of psychotropic medications for at least 30 days during the calendar year. Psychotropic polypharmacy was defined as the concurrent use of at least 2 classes of psychotropic medications. Duration of HLPP, polypharmacy, antipsychotic medication use, and psychotropic use were measured as the total number of days within the calendar year under each treatment.

### Demographic covariates

Age, sex, race, and foster care status were provided by the 2017 enrollment data file from Kentucky Medicaid. Age was categorized into two groups: children 6 to 11 years of age and 12 to 17 years. Efforts were made to supplement missing race and ethnicity data in 2017 with information from enrollment files in previous years for the same child. Race/ethnicity was constrained by the available data in Medicaid files. In Medicaid files, race/ethnicity is self-identified or identified by the parent in the case of children. A single variable was created for race and ethnicity, categorized as follows: non-Hispanic White, non-Hispanic Black, non-Hispanic Asian, non-Hispanic other (including American Indian, Native Hawaiian, and other race), and Hispanic. Metropolitan and non-metropolitan geography was determined using 2013 Rural Urban Continuum Codes (RUCC) as defined by the United States Department of Agriculture. Individuals’ geography was coded as metro when their county of residence had a RUCC code of 1 to 3 and as non-metro when their county of residence had a RUCC code of 4 to 9. Children were divided into subgroups based on foster care status.

### Statistical analysis

Demographic characteristics, utilization measures and differences in the duration of HLPP, APM, polypharmacy with at least 2 classes of psychotropic medication, and any psychotropic medication among children with at least 30 days of HLPP were summarized and presented. Comparisons between children in foster care and those in Kentucky Medicaid but not in foster care were tested using χ^2^ test for proportions, t-tests for normally distributed data, and Man-Whitney U tests for non-normally distributed continuous data, as appropriate. The prevalence of diagnoses associated with HLPP, as stratified by foster care status was compared using χ^2^ tests for proportions.

Using the reduced secondary dataset described above, a negative binomial generalized linear model was created to assess risk factors associated with longer duration of HLPP, among children with at least 30 days of HLPP. The outcome of interest was tested as a function of the demographic and diagnostic covariates discussed above. The associated incidence rate ratios are presented with 95% confidence intervals and *p* values. A 2-tailed *p* value of < 0.05 was considered statistically significant. We performed data preparation and analyses using R statistical software, version 4.4.0 (4/24/2020). The research protocol was approved by the Institutional Review Board at the University of Louisville.

## Results

### Descriptive analyses

Among children with HLPP, 19% were in foster care at some point during the calendar year. Most of the children in our sample were male, 65% among children in foster care, and 69% among children regular Medicaid. Approximately 60% of children in both groups lived in metropolitan areas. Fewer children were in the younger group (6–11 years) among children in foster care than among children in Medicaid (32% vs 40%, *p* = 0.003, Table 1). At least one instance of metabolic screening was billed for 40% of children in foster care, compared to only 27% of children in Medicaid (*p* < 0.001). Children receiving Medicaid with HLPP had more outpatient visits (*p* = 0.001) compared to children in foster care with HLPP, while emergency department and inpatient visits occurred more often among children in foster care (*p* < 0.001). No significant differences were found in the duration of HLPP, polypharmacy with at least two classes of psychotropic medication, or antipsychotic medication between children in foster care and those in regular Medicaid. Duration of any psychotropic medication was slightly higher among children in foster care (*p* < 0.01; Table 2).

**Table 1:** Demographic Characteristics of Children with High-Level Polypharmacy^a^ in 2017 by Foster Care Status Compared to Children in Medicaid

Characteristics	Children with High-Level Polypharmacy		
	Foster Care	Medicaid^b^	***p***
Number of children	417	1823	
^c^Age (years)	13.00 [11.00, 16.00]	12.00 [10.00, 15.00]	< 0.001
Age Category 6–11 years n (%)	132 (31.7)	724 (39.7)	0.003
Male n (%)	271 (65.0)	1260 (69.1)	0.115
Race/Ethnicity n (%)			< 0.001
White, non-Hispanic	206 (49.4)	1246 (68.3)	
Black, non-Hispanic	34 (8.2)	129 (7.1)	
Hispanic	9 (2.2)	29 (1.6)	
^d^Other non-Hispanic	11 (2.6)	41 (2.2)	
Not Provided	157 (37.6)	378 (20.7)	
Non-metropolitan n (%)	161 (39.1)	742 (40.8)	0.559

Note. *IQR* Intra-quartile range

^a^all children were prescribed at least 1 APM; ^b^Children receiving Medicaid who are not in foster care; ^c^ (median [IQR]); ^d^Other races/ethnicities: American Indian, Native Hawaiian, and other race

**Table 2:** Medication and resource use by children in foster care compared to those receiving Medicaid

	Children with High-Level Polypharmacy		
	Foster Care	Medicaid^a^	***p***
**Number of children**	417	1823	
^b^Pychotropic duration (days)	344.00 [315.00, 358.00]	349.00 [326.00, 358.00]	0.004
^c^Polypharmacy duration (days)	325.00 [278.00, 345.00]	330.00 [289.00, 346.00]	0.104
^d^Polypharmacy duration (days)	144.00 [67.00, 244.00]	156.00 [78.00, 255.00]	0.119
^e^APM duration (days)	285.00 [185.00, 333.00]	281.00 [181.00, 326.00]	0.193
*Utilization Measures*			
^f^Metabolic Screen (%)			< 0.001
No screen	248 (59.5)	1328 (72.8)	
1 screen	138 (33.1)	426 (23.4)	
2 screens	25 (6.0)	64 (3.5)	
3 screens	6 (1.4)	5 (0.3)	
Emergency Department Visits	2.39 (3.6)	1.72 (3.1)	< 0.001
Outpatient Visits (mean (SD))	11.09 (9.6)	14.35 (20.0)	0.001
Inpatient Visits (mean (SD))	0.81 (1.4)	0.36 (0.9)	< 0.001

Note. *IQR* Intra-quartile range

^a^Children receiving Medicaid services who are not in foster care; ^b^at least 1 psychotropic medication (median [IQR]); ^c^at least 2 psychotropic medications (median [IQR]); ^d^at least 4 psychotropic medications (median [IQR]); ^e^at least 1 antipsychotic medication (median [IQR]); ^f^Metabolic screening = presence of both lipid and glucose lab testing, as indicated by CPT codes

**Fig. 2:**
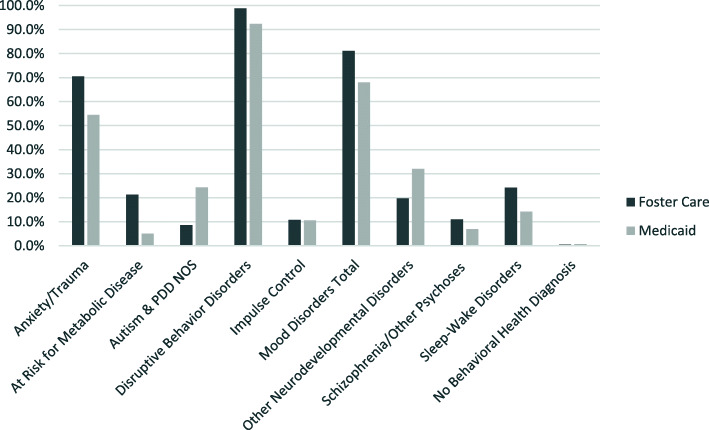
Diagnoses Associated with High-Level Polypharmacy by Foster Care Status Compared to Children Receiving Medicaid Notes: Children could have multiple diagnoses. *p* < 0.01 Schizophrenia/Other Psychoses. *p* < 0.001 Anxiety/Trauma, at Risk for Metabolic Disease, Autism & PDD NOS, Disruptive Behavior Disorders, . Mood Disorders Total, other Neurodevelopmental Disorders, Sleep-Wake Disorders. *p* > 0.05 (not significant) Impulse Control; No Behavioral Health Diagnosis

Nearly all the children in both groups were diagnosed with disruptive behavior disorders (92 and 99%). Significant proportions of children in each group were diagnosed with mood disorders (68 and 81%) and with anxiety and trauma related disorders (55 and 71%). Approximately 80% of children in both groups were diagnosed with 2 to 4 behavioral/mental health diagnoses (Fig. 2).

### Trends 2012–2017

**Fig. 3:**
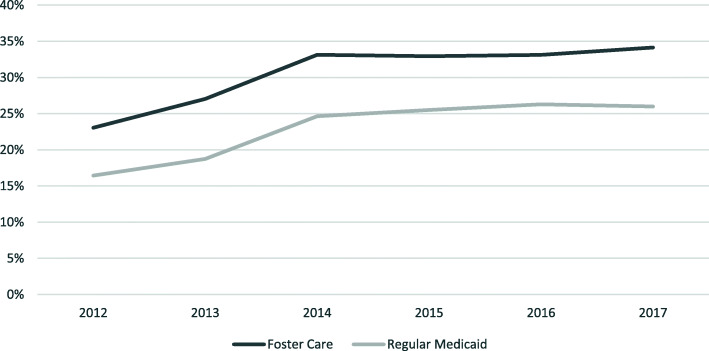
Percent of Children Receiving Kentucky Medicaid with High-Level Polypharmacy^a^ from 2012 through 2017 ^a^among children with at least one antipsychotic medication

HLPP frequency rose from 16 to 26% for children receiving Kentucky Medicaid from 2012 to 2017 with the greatest increase occurring from 2012 to 2014 (Fig. 3). In a similar pattern, children in Kentucky who are in the foster care system had rates increase from 23% in 2012 to 34% in 2017. While the use of HLPP for children in the Medicaid population began to level off in 2016, the children in foster care had a slight upward trend from 2016 to 2017. No comparison data is available related to U.S. or other states’ rates of HLPP. Although some data are available on polypharmacy, in general, the literature is inconsistent in the definitions and methods used, which makes any comparisons difficult [28, 40].

### Multivariate analyses

The duration of HLPP through the year was not significantly associated with foster care status, age, sex, race/ethnicity, or geography (Table 3). However, the presence of specific diagnoses was predictive of duration of HLPP. Disruptive behavior disorder was associated with an increase in the duration of HLPP (incidence rate ratio [IRR] = 1.18, 95% CI = [0.81, 0.99]) along with other neurodevelopmental disorders (IRR = 1.12, 95% CI = [1.04, 1.21]). A slightly shorter duration of HLPP was present for children with an impulse control disorder (IRR = 0.9, 95% CI [0.81, 0.99]).

**Table 3:** Predicting Duration of High-Level Psychotropic Polypharmacy^a^

Variables	IRR^b^	Lower Limit	Upper Limit	***p***
Residence - Metro	***ref***			
Non-metro	1.02	0.96	1.09	0.462
Sex - Female	***ref***			
Male	1.04	0.97	1.11	0.268
Age Category - 12-17 years	***ref***			
6–11 years	0.96	0.9	1.02	0.196
Race/Ethnicity – White, Non-Hispanic	***ref***			
Black, Non-Hispanic	1.09	0.98	1.22	0.097
Hispanic	1.05	0.86	1.31	0.641
Other	0.92	0.77	1.1	0.341
Foster Care	0.96	0.88	1.05	0.319
*Diagnostic Categories*				
Schizophrenia and Other Psychotic Processes	0.95	0.84	1.08	0.446
Mood Disorders Total	0.96	0.89	1.03	0.259
Autism	1.06	0.98	1.15	0.153
Other Neurodevelopmental Disorders	**1.12**	**1.04**	**1.21**	**0.002**
Disruptive Behavior Disorders	**1.18**	**1.04**	**1.34**	**0.011**
Anxiety and Trauma Related Disorders	0.95	0.89	1.01	0.118
Impulse Control	**0.9**	**0.81**	**0.99**	**0.030**
Sleep-Wake Disorders	0.92	0.85	1.01	0.069

^a^among children with at least one antipsychotic medication prescription and at least 30 days of high-level psychotropic polypharmacy; ^b^incidence rate ratio (IRR)

## Discussion

The use of HLPP has expanded significantly for both children in Medicaid and those in foster care over the years 2012–2017 in Kentucky. Previous studies have shown recent declines in the rates of APM use in children [41, 42]. Our study joins others in suggesting that the practice of concomitant medication use continues to grow in the treatment of children on Medicaid and in foster care [5, 8, 39, 43, 44] despite efforts to safeguard their use.

Concerns over the overutilization of psychotropic medications in children in foster care has led to the development of federal and state policies to promote greater oversight of psychotropic medication use in children and youth and now almost all states employ some mechanism to monitor psychotropic medication use in children in foster care [9, 10, 18, 45, 46]. In addition, many states have programs that prospectively or retrospectively monitor psychotropic medications for all children on Medicaid [29]. Most existing psychotropic medication monitoring programs focus on single psychotropic medications [5, 47, 48]. At the time of our study, Kentucky had no program in place to monitor psychotropic medication use for children in foster care, so oversight would have been addressed by existing managed care program policies. Kentucky Medicaid is administered through managed care organizations. Our study suggests the need for psychotropic medication programs that target HLPP.

Our study suggests that both children in foster care and those in Medicaid are susceptible to HLPP and that there were not many differences between the two populations in our study. Children on Kentucky Medicaid had a slightly longer duration of HLPP, but the difference in duration was not clinically significant. Children in foster care on HLPP tended to be slightly older than their counterparts receiving Medicaid who were not in foster care.

In our sample, a minority of children received a laboratory test for cholesterol and a glucose or A1C during 2017, which quality metrics recommend [34, 49, 50]. Others have similarly reported poor monitoring [48, 51]. While metabolic screening remains poor in both groups, children in foster care received metabolic screening more often than their peers, suggesting greater monitoring. However, children in foster care also had more claims for emergency department visits and inpatient hospitalizations. It is uncertain whether these findings suggest over-utilization or greater oversight. Children in foster care had fewer outpatient visits, but this finding is obscured by the fact that about 50% of children in foster care in Kentucky have bundled service payment models with private childcare agencies, which does not result in a claim being submitted for some services such as psychosocial therapy. Improved methods of increasing the rates of metabolic monitoring for children on an antipsychotic medication and HLPP is needed since concomitant use of antidepressants and APM have been shown to increase the risk of Type 2 diabetes mellitus [44].

It has been shown that polypharmacy is more common in families at risk for socioeconomic adversities and disabilities [5, 52] and more attention is needed to ensure these families and vulnerable youth receive evidence-based psychosocial therapy prior to medication initiation [52, 53]. Once a child enters foster care, he/she may be more likely to have psychosocial therapy and preventive health monitoring, but that could not be determined from the current dataset.

Diagnoses predictive of HLPP, in our sample included other neurodevelopmental disorders (intellectual disabilities, tic disorders, movement disorders, and developmental disorders), disruptive behavioral disorders (attention-deficit hyperactivity disorder, oppositional defiant disorder, conduct disorder) and impulse control disorders (intermittent explosive disorder). Others have suggested that antipsychotic medications are increasingly used to treat disruptive behaviors, especially for children in foster care [29]. Surprisingly, foster care status did not predict the duration of HLPP. One explanation could be that receiving an antipsychotic medication was an inclusion criterion. There were no demographic predictors of duration of HLPP use.

Many concerns exist for the continuing practice of prescribing HLPP. Evidence is lacking related to the safety and efficacy of the use of multiple medications in children and youth [5, 7, 54]. Keider and colleagues suggested that further research is needed to determine the efficacy of these drug combinations and to identify potential drug-to-drug interactions among various combination therapies [5]. They go on to suggest that evidence-based psychosocial therapies are not being used adequately [33].

Antipsychotic medications are commonly used in HLPP [55]. Of particular concern is that the use of antipsychotic medication in children and youth is associated with many significant side effects such as weight gain, Type 2 diabetes mellitus, and lipid abnormalities [31, 50]. In addition, basic patterns of treatment for common conditions may be changing. For example, 47.9% of children recently diagnosed with ADHD did not receive a stimulant prior to an APM [55]. Our study joins others to raise concerns over the practice of high-risk pharmacotherapy [5, 21, 29, 55–57].

Children receiving Medicaid services and those in foster care are particularly vulnerable populations. However, these systems also provide opportunities to implement policies to guide safe prescribing and appropriate monitoring [58]. Some interventions have included prior authorizations, retrospective chart reviews of prescribing practices, learning collaborative, and greater oversight by child and adolescent psychiatrists [29, 58].

This study is not without limitations. There are known limitations in using administrative claims data [59]. Prescribing is only captured if a claim is submitted, which could lead to under reporting of actual prescribing [59]. A provider may omit submitting claims for services that are generally not reimbursed or that are reimbursed at a rate deemed too low for the effort required. The practice of “bundling” payment for groups of services may result in unfiled claims. The bundling of services occurs for some children in foster care, such as those in residential facilities. The bundling of payments generally does not occur with medications but, rather, with services such as psychosocial therapy. In the case of the current study, outpatient visits could be under-represented for a child receiving a well-child examination within a residential facility. Certain details of the disease characteristics and/or the treatment type or quality are not provided in claims data [59]. Another limitation is that race/ethnicity is an optional field in the claims data resulting in missing data and the racial/ethnic designations are limited. More granular analysis of race/ethnicity is not possible with the current dataset. Lastly, the data represent only one state, so it is not known whether the findings are generalizable to other states.

Medical and pharmacy claims data do not allow for analyses to examine the accuracy of the diagnosis, the appropriateness of the medications or other treatment types nor do they allow for determinations of provider bias in diagnosis and treatment types. While it is known that structural racism exists in welfare and healthcare systems, prospective data collection is needed to examine the effects of these social determinants of health on the treatment of children’s mental health problems [60–63].

Despite the limitations, the current study presents new information related to concerning patterns of HLPP that includes at least one antipsychotic medication for children in foster care and their peers who are receiving Medicaid services. With little evidence for the safety and efficacy of these medication combinations, more research and quality improvement efforts are needed to ensure that evidence-based practices are being developed and implemented to safeguard the use of psychotropic medications, especially in our most vulnerable children such as those living in poverty and those in foster care.

## Summary

Our study identifies increasing rates of HLPP from 2012 to 2017 in our sample of children in foster care and those receiving Medicaid who are also on an antipsychotic medication. Clinical diagnoses with features of disruptive and impulsive behaviors predicts HLPP use. Metabolic screening rates in our population are low. There is a need for improved oversight of HLPP in both children in foster care and those receiving Medicaid to ensure that these vulnerable children are receiving comprehensive mental and physical health services with a focus on minimizing pharmacotherapy to the least amount necessary to promote optimal child functioning [5]. Federal and state policies have been implemented in recent years to require states to have oversight plans for psychotropic medication use for children in foster care [29]. Such plans should include prior authorization policies focusing specifically on HLPP. Prior authorization programs have the most evidence for efficacy in reducing the use of antipsychotic medications [64]. More data are needed to determine if prior authorization is the most effective intervention for reducing the use of other medication and for polypharmacy. Other system-level approaches have been recommended to improve the quality and availability of psychosocial therapies as an alternative to using psychotropic medications, using the electronic health record reminders and best-practice alerts, improved care coordination, and improving access to rapid consultation with child mental health providers [29].

## Supplementary Information

**Additional file 1.**

**Additional file 2.**

## Data Availability

The data used for this study are not publicly available. The data belong to the Kentucky Cabinet for Health and Family Services, Department for Medicaid Services. The authors had a contractual agree to use these data from a State-University Partnership Program. Request for data use should be directed to the Kentucky Cabinet for Health and Family Services, Department for Medicaid Services, 275 E Main St, Frankfort, Kentucky 40601 USA.
