# Supplementary material for: High-level psychotropic polypharmacy: a retrospective comparison of children in foster care to their peers on Medicaid
Source: BMC Psychiatry. 2021 Jun 10;21:303. doi: 10.1186/s12888-021-03309-9 (PMC8194140; doi:10.1186/s12888-021-03309-9)
Supplement: Supplementary file 1 — Additional file 1. [file 12888_2021_3309_MOESM1_ESM.docx]

| **Table S1 (Supplemental): Codes and Categories for Diagnoses and Procedures** | |  | |  |
| --- | --- | --- | --- | --- |
| **Categories of Diagnoses and Procedure Codes (CPT) Included** |  | | |  |
|  | **CPT Codes** | | |  |
| **Outpatient** | 99201-99205, 99211-99215, 99241-99245 | | |  |
| **Emergency** | 99281-99285 | | |  |
| **Inpatient** | 99221-99223 | | |  |
| **Lipids** | 80061, 82465, 83718, 84478, 83700, 83701, 83704, 83721 | | |  |
| **Glucose** | 82947, 82950, 82951, 83037 | | |  |
| **Metabolic Screening** | Lipids and Glucose | | |  |
|  | **ICD-9 Codes** | **ICD-10 Codes** | |  |
| **Schizophrenia and Other Psychotic Processes** |  | |  | |
| Schizophrenia and Schizoaffective Disorder | 295.xx | | F20, F200-F205, F208, F2081,F2089, F209, F25.0, F25.1 | |
| Delusional Disorders | 297.x | | F22 | |
| Other Nonorganic Psychoses | 298.x | | F28, F23, F29 | |
|  |  | |  | |

| **Mood Disorders Total** |  |  |
| --- | --- | --- |
| Depressive Disorders (listed below) | 296.20-296.36, 300.4, 311, 625.4 | F32.0 - F32.5, F32.8, F32.9, F33.0- F33.3, F33.8, F33.9, F33.41, F33.42, F34.1, N94.3 |
| Bipolar and Related Disorders | 296.00-296.10, 296.40-296.89,and 301.13 | F30.10-F30.4, F30.8, F30.9, F31.0-F31.9, F34.0 |
| Mood Disorder NOS | 296.9 | F06.3, F39 |
| Disruptive Mood Dysregulation Disorder | 296.99 | F34.8 |
|  |  |  |
| **Autism Spectrum Disorders** | 299.xx | F80.89, F84, F840, F842, F843, F845, F848, F849 |
|  |  |  |
| **Other Neurodevelopmental Disorders** |  |  |
| Intellectual Disability | 317.xx, 318.xx, 319.xx, 758 | Q909, F70-F73, F78, f79 |
| Tic Disorders | 307.20-307.23 | F95, F950-F952, F958, F959 |
| Stereotypical Movement Disorder | 307.3 | F984 |
| Developmental Delays | 315.xx | F80, F80.81, F80.9, F81, F81.1, F81.2, F82, F88, F89 |
| **Disruptive Behavior Disorders** |  |  |
| Conduct Disorders | 312.81, 312.89, 312.90 | F91x except F913 |
| Attention Deficit Disorder | 314.xx | F90x |
| Oppositional Defiant Disorder | 313.81 | F913 |
|  |  |  |
| **Anxiety and Trauma Related Disorders** |  |  |
| Anxiety Disorders | 300.00-300.29, 309.21, 301.4, and 312.23 | F40-F419, F93.0, F94.0 |
| PTSD and Acute Stress Disorder | 308.3, 309.81, 309.89, 309.9 | F430, F431-F4312, F43.8 |
| OCD and Related Disorders | 300.3, 300.7, and 312.39 | F42, F45.22, F63.2, L98.1 |
| Adjustment Disorders | 309.0, 309.24, 309.28, 309.3, 309.4, 309.89, 309.9 | F43, F432-F439 |
| Reactive Attachment Disorder | 313.89 | F938, F941, F942, F988 |
|  |  |  |
| **Impulse Control** |  |  |
| Intermittent Explosive Disorder and Impulse Control | 312.30 - 312.34, 312.89, and 312.9. | F63-F632, F633, F638-F639 |
|  |  |  |
| **Sleep-Wake Disorders** | 780.52, 780.54, 307.45, 307.46, 307.47, 327.21, 327.23, 327.42, 333.94, 347.00, 347.01, 347.10 | F51, F510, F5101 - F5105, F5109, F511, F5111-F5113, F5119 F513-F515, F518, F519, G25.81, G47.00, G47.09, G47.10, G47.19, G47.20 - G47.26, G47.31, G47.33, G47.411, G47.419, G47.429, G47.52, G47.8 |
|  |  |  |
| **Insulin Resistance** | 259.8 | E348 |
| **Type II Diabetes** | 250.00, 250.02, 250.40, 250.42, 250.50, 250.52, 250.60, 250.62, 250.70, 250.72, 250.80, 250.82, 250.90, 250.92 | E08, E09, E11, E13 |
| **Pre-Diabetes** | 790.21, 790.22 | R7301, R7302 |
| **Hyperlipidemia** | 272.00-272.90, 288.00, 285.90 | E780-E789 |
| **At Risk for Metabolic Disease** | At least one of insulin resistance, type II diabetes, pre-diabetes, or hyperlipidemia | |
|  |  |  |
| **Physical Diagnostic Categories** |  |  |
| Seizure Epilepsy | 345.XX | G40 |
| Other Convulsions | 780.39 | R56.9 |
| Post Traumatic seizures |  | R56.1 |
