# Supplementary material for: High-level psychotropic polypharmacy: a retrospective comparison of children in foster care to their peers on Medicaid
Source: BMC Psychiatry. 2021 Jun 10;21:303. doi: 10.1186/s12888-021-03309-9 (PMC8194140; doi:10.1186/s12888-021-03309-9)
Supplement: Supplementary file 2 — Additional file 2. [file 12888_2021_3309_MOESM2_ESM.docx]

**Table S2 (Supplemental): Medication Classes**

| **Antipsychotics** | | | |
| --- | --- | --- | --- |
| Abilify | Fluphenazine Decanoate | Olanzapine ODT | Risperdal |
| Abilify Discmelt | Fluphenazine HCL | Olanzapine-Fluoxetine HCL | Risperdal Consta |
| Abilify Maintena | Geodon | Orap | Risperdal M-Tab |
| Amitriptyline HCL/Perphenazine | Haldol | Paliperidone | Risperidone |
| Amitriptyline/Perphenazine | Haldol Decanoate 100 | Paliperidone (Invega) | Risperidone ODT |
| Amtriptyline Perphenazine | Haloperidol | Perphenazine | Saphris |
| Aripiprazole | Haloperidol Decanoate | Perphenazine & Amitriptyl | Seroquel |
| Aripiprazole (Abilify) | Haloperidol Decanoate 100 | Perphenazine 2mg W/Amitri | Seroquel XR |
| Aripiprazole ODT | Haloperidol Lactate | Perphenazine 4mg W/Amitri | Thioridazine |
| Asenapine | Iloperidone | Perphenazine Amitriptylin | Thioridazine HCL |
| Asenapine (Saphris) | Iloperidone (Fanapt) | Perphenazine And Amitript | Thiothixene |
| Chlorpromazine | Invega | Perphenazine W/Amitrip | Trifluoperazine |
| Chlorpromazine HCL | Invega Sustenna | Perphenazine/Amitrip | Trifluoperazine HCL |
| Clozapine | Invega Trinza | Perphenazine-Amitriptyline | Ziprasidone |
| Clozapine ODT | Latuda | Prochloperazine | Ziprasidone HCL |
| Clozaril | Loxapin | Prochlorperazine | Zyprexa |
| Fanapt | Loxapine | Prochlorperazine Maleate | Zyprexa Zydis |
| Fazaclo | Lurasidone | Quetiapine |  |
| Fluphenazine | Olanzapine | Quetiapine Fumarate |  |

| **Moodstabilizers** | | | |
| --- | --- | --- | --- |
| Carbamazepine | Equatro | Lamotrigine ER | Oxtellar |
| Carbamazepine ER | Equetro | Levetiracetam | Oxtellar XR |
| Carbamazepine XR | Eskalith | Levetiracetam ER | Tegretol |
| Carbatro | Gabapentin | Lithane | Tegretol XR |
| Carbatrol | Gralise | Lithium | Topamax |
| Cibalith-S | Keppra | Lithium Carbonate | Topamax |
| Depakene | Keppra XR | Lithium Carbonate ER | Topiram |
| Depakote | Lamictal | Lithium Citrate | Topiramate |
| Depakote ER | Lamictal (Green) | Lithobid | Topiramate ER |
| Depakote Sprinkle | Lamictal (Orange) | Lithonate | Trileptal |
| Divalproex | Lamictal ODT | Lithotabs | Trokendi XR |
| Divalproex Sodium | Lamictal ODT (Green) | Lyrica | Valproate |
| Divalproex Sodium ER | Lamictal ODT (Orange) | Magnesium Sulfate | Valproate Sodium |
| Epitol | Lamictal XR | Neurontin | Valproic Acid |
| Epsom Salt | Lamotrigine | Oxcarbazepine |  |

| **Antidepressants** | | | |
| --- | --- | --- | --- |
| Adapin | Desipramine HCL | Luvox CR | Savella |
| Alplenzin | Desvenlafaxine | Maprotiline | Selegiline |
| Amitril | Desyrel | Maprotiline HCL | Sertraline |
| Amitrip HCL/Chlordiazepoxide | Doxepin | Marplan | Sertraline HCL |
| Amitriptyline | Doxepin HCL | Milnacipran | Serzone |
| Amitriptyline HCL | Duloxetine | Mirtazapine | Silenor |
| Amoxapine | Duloxetine HCL | Nardil | Sinequan |
| Anafranil | Effexor | Nefazodone | Sk-Amitriptyline |
| Asendin | Effexor XR | Nefazodone HCL | Sk-Pramine |
| Aventyl | Elavil | Norpramin | Surmontil |
| Budeprion | Emsam | Nortriptyline | Symbyax |
| Budeprion SR | Endep | Nortriptyline HCL | Tofranil |
| Budeprion XL | Escitalopram | Oleptro | Tofranil-PM |
| Buproban | Escitalopram (Lexapro) | Oleptro ER | Tranylcypromine Sulfate |
| Bupropion | Escitalopram Oxalate | Pamelor | Trazodone |
| Bupropion HCL | Etrafon | Parnate | Trazodone HCL |
| Bupropion HCL SR | Fluoxetine DR | Paroxetine | Triavil |
| Bupropion Xl | Fluoxetine HCL | Paroxetine HCL | Trimipramine Maleate |
| Cdp & Amitriptyline | Fluvoxamine | Paxil | Tryptanol |
| Celexa | Fluvoxamine Maleate | Pertofrane | Venlafaxine |
| Chlordiazepoxide And Amitrip | Fluvoxamine Maleate ER | Phenelzine Sulfate | Venlafaxine HCL |
| Chlordiazepoxide/Amitrip | Imipramine | Presamine | Venlafaxine HCL ER |
| Chlordiazepoxide-Amitriptyline | Imipramine HCL | Pristiq | Viibryd |
| Chlordiazepoxide-Clidinium | Imipramine Pamoate | Pristiq ER | Vilazodone |
| Citalopram | Isocarboxazid | Protriptyline HCL | Vivactil |
| Citalopram Hbr | Janimine | Prozac | Wellbutrin |
| Clomipramine HCL | Lexapro | Remeron | Wellbutrin XL |
| Clomipramine Hydrochloride | Limbitrol | Sarafem | Xenazin |
| Cymbalta | Ludiomil | Sarna | Xenazine |
| Desipramine | Luvox | Sarna Sensitive | Zoloft |

| **Benzodiazepines-Anxiolytics-Hypnotics** | | | |
| --- | --- | --- | --- |
| Alprazolam | Chlordiazepoxide HCL | Hydroxyzine HCL | Phenobarbital |
| Alprazolam ER | Clorazepate Dipotassium | Hydroxyzine Pamoate | Rozerem |
| Alprazolam ODT | Diastat | Lorazepam | Temazepam |
| Alprazolam XR | Diastat Acudial | Lorazepam Intensol | Triazolam |
| Ambien CR | Diazepam | Lunesta | Zaleplon |
| Ativan | Eszopiclone | Midazolam HCL | Zolpidem Tartrate |
| Buspirone HCL | Flurazepam HCL | Oxazepam | Zolpimist |

| **Alpha-2-Agonists** | | | |
| --- | --- | --- | --- |
| Catapres | Clonidine | Guanfacine HCL | Methyldopa |
| Catapres-TTS 1 | Clonidine HCL | Guanfacine HCL ER | Methyldopa -Hydrochlorothiazide |
| Catapres-TTS 2 | Clonidine HCL ER | Intuniv |  |
| Catapres-TTS 3 | Guanfacine | Kapvay |  |

| **Lithium** | | | |
| --- | --- | --- | --- |
| Cibalith-S | Lithium | Lithium Citrate | Lithotabs |
| Eskalith | Lithium Carbonate | Lithobid |  |
| Lithane | Lithium Carbonate ER | Lithonate |  |

| **Stimulants** | | | |
| --- | --- | --- | --- |
| Adderall | Dexmethylphenidate | Metadate | Quillivant |
| Adderall XR | Dexmethylphenidate HCL | Metadate CD | Quillivant XR |
| Amphet ASP/Amphet/D-Amphet | Dexmethylphenidate HCL ER | Metadate ER | Ritalin |
| Amphetamine Salt Combo | Dextroamphetamine | Methamphetamine HCL | Ritalin LA |
| Amphetamine /Dextroamphetamine | Dextroamphetamine Sulfate | Methylphenidate | Ritalin-SR |
| Atomoxetine | Dextroamphetamine Sulfate ER | Methylphenidate ER | Strattera |
| Concerta | Dextroamphetamine -Amphet ER | Methylphenidate HCL | Vynase |
| Cylert | Dextrostat | Methylphenidate HCL CD | Vyvanse |
| D-Amphetamine Sulfate | Focalin | Methylphenidate LA | Dextroamphetamine- Amphetamine |
| Daytrana | Focalin XR | Methylphenidate SR |  |
| Desoxyn | Lisdexamfetamine | Pemoline |  |
| Dexedrine | Lisdexamfetamine (Vynase) | Procentra |  |
